# The Protective Effects of Vanillic Acid and Vanillic Acid-Coated Silver Nanoparticles (AgNPs) in Streptozotocin-Induced Diabetic Rats

**DOI:** 10.1155/2024/4873544

**Published:** 2024-03-28

**Authors:** Eman S. Alamri, Haddad A. El Rabey

**Affiliations:** ^1^Department of Nutrition and Food Science, University of Tabuk, Tabuk 47512, Saudi Arabia; ^2^Biochemistry Department, Faculty of Science, University of Tabuk, Tabuk 47512, Saudi Arabia

## Abstract

The production of nanoparticles enhances the bioactivity of biological molecules for drug delivery to diseased sites. This study explains how silver nanoparticle (AgNP) coating enhanced the protection effects of vanillic acid in male diabetic rats with streptozotocin- (STZ-) induced diabetes. Twenty-four rats were divided into four groups (*n* = 6) for this investigation. The first group (G1) is untreated, whereas diabetes was induced in the other three groups through STZ injection. Diabetic rats that were not getting therapy were included in the second group (G2, STZ-positive), whereas the other diabetic rats were divided into the third group (G3, vanillic acid-treated) and the fourth group (G4, vanillic acid-coated AgNPs treated). The treatment lasted four weeks. In G2, the induction of diabetes significantly (at *P* = 0.05) increased in serum glucose, glycated proteins, renal indices, interleukin-6 (IL-6), K+, immunoglobulins, and lipid peroxidation, while decreased Ca++, Na+, and other antioxidants in the kidney tissue homogenate. In addition, pathological altered signs were present in the pancreas and kidneys of diabetic rats. The renal and pancreatic tissues were effectively enhanced by vanillic acid or vanillic acid-coated AgNPs, bringing them very close to their prediabetic conditions. Vanillic acid-coated AgNPs offered a stronger defense against STZ-induced diabetes and lessened the effects of hyperglycemia compared to ordinary vanillic acid. Additionally, using vanillic acid coated with silver nanoparticles greatly increased the antioxidant and antidiabetic activity and reduced inflammation when compared to using vanillic acid alone.

## 1. Introduction

In type 1 diabetes mellitus, less insulin is generated, whereas in type 2, the body has problems utilizing the insulin that is produced [1]. It is predicted that there will be 592 million people worldwide with diabetes mellitus making it one of the top 10 killers worldwide [2]. Serious macro- and microvascular consequences of diabetes mellitus are caused by persistently insufficient glycemic control [3]. Diabetes is not explicitly treated in the modern medical system, since angiotensin-converting enzyme inhibitors and antidiabetic medications can be used alone or in combination to treat it early and stop the problem from progressing to an overt stage of nephropathy [4].

Many antidiabetic natural products were used to treat diabetes such as vanillic acid—a flavoring phenolic agent with antioxidant properties that has been successfully used in treating a variety of diseases brought on by oxidative stress and reactive oxygen species (ROS), including CCl_4_-induced liver and kidney toxicity and streptozotocin-induced diabetic neuropathy [5–7]. Vanillic acid and Zn(II) complexes are also used as antioxidants and antidiabetics [8]. In addition, in streptozotocin-induced diabetic rats, it reduced the oxidative stress and inflammation brought on by diabetes [9].

The antioxidant activity of silver nanoparticles (AgNPs) of vanillic acid improved the treatment of diseases brought on by excessive oxidative stress [10, 11]. Metallic and polymer nanoparticles created biologically were used for diagnostic purposes and to increase the effectiveness of medicine delivery [10, 12–14].

Silver nanoparticles were also extensively used in the biomedical sector [15], in the development of nanomedicine and for numerous pharmacological functions [16]. Silver nitrate and imipenem nanoparticles successfully battled off *Pseudomonas aeruginosa*, a drug-resistant bacterium [17]. Additionally, *Staphylococcus aureus* was shielded from building a vancomycin resistance by the silver nanoparticles. Furthermore, encasing infectious germs in nanoparticles made them more vulnerable to drugs; for instance, fluconazole and biogenic-loaded silver nanoparticles were employed to make the drug-resistant *Candida* spp. more sensitive to fluconazole [18]. Additionally, curcumin-based nanoformulations were added to fungal chitosan nanoparticles, increasing their anticancer potency [19].

This study sets out to evaluate whether vanillic acid might have a hypoglycemic effect and whether AgNP coating might improve that hypoglycemic effect.

## 2. Materials and Methods

### 2.1. Chemicals and Animals

All of the chemicals, reagents, and kits used in this inquiry were analytical grade and purchased from Sigma-Aldrich, USA, unless another resource was mentioned (CAS number of vanillic acid is 121-34-6). However, the Sprague Dawley rats were supplied by the Giza Agricultural Research Center in Egypt.

### 2.2. Preparation of Vanillic Acid-Coated AgNPs

Color-mediated silver ion reactions were used to create vanillic acid, which was then coated with silver nitrate nanoparticles [10, 11]. Two grams of vanillic acid was dissolved in a few drops of ethanol before being combined with 100 ml of distilled water. Then, 20 mg of NaNO_3_ was added, mixed with a stirrer, and then added to get the pH to 10.0. The color of the mixture was changed from colorless to yellow to dark brown, ensuring the formation of silver nanoparticles [10].

### 2.3. Assessment of the Properties of Synthesized Vanillic Acid-Coated AgNPs

#### 2.3.1. Visualization of the Vanillic Acid-Coated AgNPs under Transmission Electron Microscopy (TEM)

The produced vanillic acid-coated AgNP size, shape, assembly, and purity were all visualized and analyzed using a transmission electron microscope. The sample's TEM grids were cleaned, cleared out with filter paper, and then placed in a few drops, each around 2 to 5 *μ*l in volume, on a parafilm sheet [10, 20].

#### 2.3.2. Examination of the Synthesized Vanillic Acid-Coated AgNPs Using UV-Visible Spectroscopy

To identify the surface plasmon resonance of the synthesized vanillic acid-coated AgNPs, the UV-vis spectra of the synthesized vanillic acid nanoparticles were examined between 300 and 800 nm [10, 21].

### 2.4. Dynamic Light Scattering (DLS) of the Synthesized Vanillic Acid-Coated AgNPs

The size and distribution peak of the produced vanillic acid-coated AgNPs were measured using the dynamic light scattering (DLS) method. Before analysis, the produced vanillic acid AgNPs were diluted ten times with deionized water. Then, in a clean cuvette, 25 *μ*l of the produced vanillic acid-coated AgNPs was combined, equilibrated for 2 minutes at 20°C, dispersed, and scaled using the particle sizing device “NICOMP Nano ZLS (Z3000 zls)” (Entegris, Germany) [5, 22].

### 2.5. Experimental Methods and Animals

24 male rats (Sprague Dawley, weighing 180.0 ± 10.0 g) which the study's animals were purchased from the Agricultural Research Center, Giza, Egypt, and housed for 14 days in standardized lab conditions at the Faculty of Pharmacy at Mansoura University, which has an approved animal house ethics program (code: 0185 in 20-05-2022). In this study, we used male rats to avoid bias caused by estrogen. Female estrogens protect rats against oxidative stress, by inducing antioxidant genes that will affect the results of the experiment and interfere with the treatment materials [23]. Throughout the experiment, food and water were readily available. The rats were divided into four groups (*n* = 6 rats) of rats after acclimation. The first group (G1) was the negative control group that received one dose of 0.1 mol/l citrate buffer (pH 4.5) injected in the tail vein. The remaining rats were fasted for 12 hours before receiving an intravenous injection of streptozotocin (65 mg/kg bw) in a freshly made, 0.1 mol/l citrate buffer (pH 4.5) [24]. The rats are considered diabetic if blood glucose measurements show a level greater than 200 mg/dl. The diabetic rats were divided into three groups at random: the second group (G2) was left untreated as a control-positive diabetic group, the third group (G3) was treated daily with vanillic acid (100 mg/g bw) using stomach gavage at 9:00 a.m., and the fourth group (G4) received daily treatment with vanillic acid-coated AgNPs (100 mg/g bw) using stomach gavage at 9:00 a.m. [25]. The treatment was conducted for four weeks after the induction of diabetes.

### 2.6. Dissection and Blood Collection

At the end of the treatment period (four weeks), the rats fasted for 12 hours before being euthanized in their cages using carbon dioxide (by displacing 30% to 70% of the cage volume per minute by carbon dioxide flow) which caused narcosis; cervical dislocation was achieved and then dissected. One kidney and the pancreas were washed with saline solution and kept in 10% formalin, whereas the other kidney was kept on ice to prepare kidney tissue homogenate. Blood was collected from the heart of the sedated animals, centrifuged for five minutes at 3000 rpm to separate the plasma, then transferred to clean tubes, and kept in the fridge for biochemical analysis.

### 2.7. Preparation of Renal Tissue Homogenate

The kidney tissue was homogenized in an ice-cold phosphate buffer (pH 7.4). The mixture was centrifuged at 4000 rpm for 15 minutes, and the resulting supernatant was collected and used to determine the amount of lipid peroxidation and antioxidant enzymes.

### 2.8. Biochemical Analyses

#### 2.8.1. Kidney Function

Human Diagnostic Kits (Germany) were used to measure the levels of urea, creatinine, uric acid, potassium (K+), calcium (Ca++), and sodium (Na+) ions in blood serum.

#### 2.8.2. Interleukin-6 (IL-6)

Serum interleukin-6 (IL-6) was determined using the MyBioSource Kit (San Diego, USA).

#### 2.8.3. Estimation of Fasting Blood Sugar and HbA1c

The fasting blood sugar was estimated according to the method of Trinder [26] using Human Kit (Germany), whereas the HbA1c was estimated in whole blood using VARIANT II Hemoglobin Testing System (USA). All analyses were done according to the instructions of the suppliers.

#### 2.8.4. Immunoglobulins

The GenWay Biotech Kit (USA) was used by the manufacturer's instructions to calculate immunoglobulins (IgG, IgA, and IgM) using Berne's method [27].

#### 2.8.5. Antioxidants and Lipid Peroxidation

The Biodiagnostic Kit (Egypt) was used to assess the activity of superoxide dismutase (SOD), glutathione-s-transferase (GST), and catalase (CAT) in the kidney tissue homogenate, according to Habig et al. [28], Nishikimi et al. [29], and Aebi [30], respectively. Malondialdehyde (MDA) in the kidney tissue homogenate was also assessed using the same biodiagnostic method.

### 2.9. Histopathology

The 10% formalin-fixed kidney and pancreatic tissues underwent paraffin embedding, ethanol dehydration (in a 70, 80, and 90% series), and xylene clearing. 5 *μ*m slices were cut into microtome sections and stained with hematoxylin and eosin (H&E) [31].

### 2.10. Statistical Analysis

The Statistical Package for the Social Sciences (SPSS) software, version 17.0, was used for data analysis [32]. The data are presented as mean ± standard deviation. Testing the differences between groups was calculated using a one-way analysis of variance (post hoc Duncan).

## 3. Results

### 3.1. AgNP-Coated Vanillic Acid

**Figure 1:**
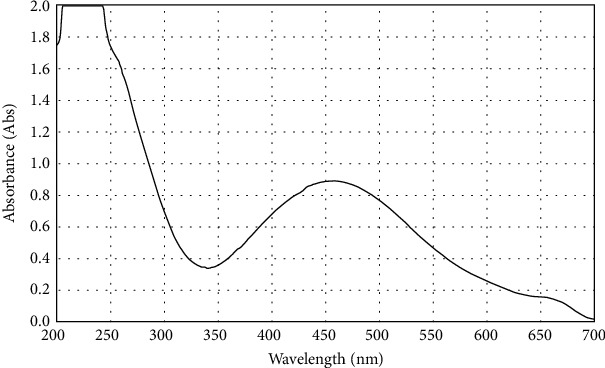
UV-visible spectra displaying the peak of the surface plasmon resonance of vanillic acid-coated AgNPs at 450 nm.

**Figure 2:**
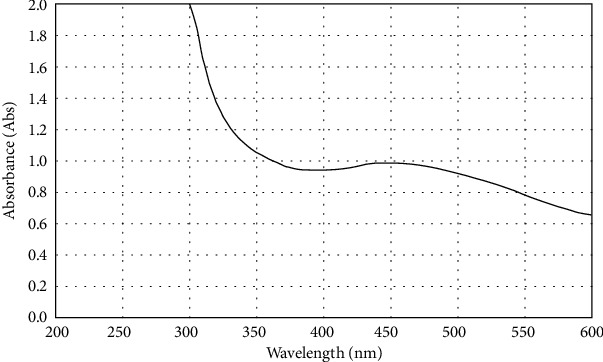
UV-visible spectrum displays the peak of the surface plasmon resonance of AgNPs at about 450 nm.

The color of the vanillic acid solution changed to dark brown after adding silver nitrate to let the AgNPs coat vanillic acid, indicating the formation of the silver nanoparticle. The obtained substance's UV-vis spectrum was examined to confirm the creation of surface plasmon resonance (SPR) peak for vanillic acid-coated AgNPs at 450 nm wavelengths (Figure 1), whereas the UV-visible spectra show a peak of the surface plasmon resonance of the AgNPs at about 450 nm as shown in Figure 2. The wavelength of the synthesized vanillic acid-coated AgNPs showed that the maximum UV-vis absorption between 400 and 500 nm ensured that the AgNPs and the vanillic acid-coated AgNPs were generated during biosynthesis.

**Figure 3:**
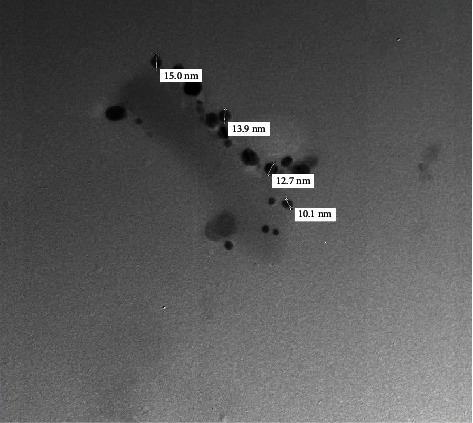
TEM image of the vanillic acid-coated AgNPs; the particles ranged in size from 10.1 to 15.0 nm.

**Figure 4:**
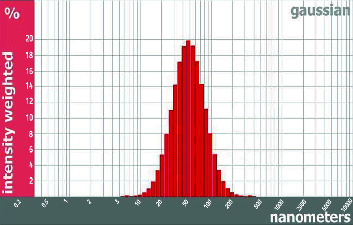
The synthesized vanillic acid-coated AgNPs have an average particle size of 52.3 nm, according to the DLS analysis.

Figure 3 displays the TEM image of the vanillic acid-coated AgNPs of a size ranging from 10.1 to 15.0 nm [10, 20]. In addition, according to the results of the dynamic light scattering (DLS) investigation shown in Figure 4, the generated vanillic acid-coated AgNPs had an average size of 52.3 nm.

### 3.2. FBS, HbA1c, and IL-6

Table 1 displays the effects of administering vanillic acid and vanillic acid-coated AgNPs in STZ-induced diabetic rats on FBS, HbA1c, and IL-6. G2 rats had greater blood levels of FBS and HbA1c than G1 rats due to STZ-induced diabetes. In contrast, diabetic rats in G3 and G4 treated with vanillic acid or vanillic acid-coated AgNPs had significantly lower blood levels of FBS, HbA1c, and IL-6. The increased levels of FBS, HbA1c, and IL-6 were more effectively reduced by treatment with vanillic acid-coated AgNP vanillic acid in G4 than by treating with ordinary vanillic acid in G3.

### 3.3. Antioxidant and Lipid Peroxidation

Table 2 illustrates the effect of STZ-induced diabetes on antioxidants and lipid peroxidation of renal tissue homogenate. Decreased values of GST, SOD, and CAT were estimated in G2 when hyperglycemia was induced. The elevated levels of malondialdehyde (MDA) in the kidney tissue homogenate indicate that lipid peroxidation was also elevated. Following vanillic acid therapy, the antioxidant enzyme was somewhat increased and lipid peroxidation was decreased in G3. Whereas when diabetic rats were treated with vanillic acid-coated AgNP3, the antioxidant enzymes were significantly increased and the degree of lipid peroxidation was decreased in G4.

### 3.4. Kidney Function and Serum Electrolytes

In G2, STZ-induced diabetes was associated with significantly higher levels of urea, creatinine, uric acid, and potassium ions (K+) and lower levels of sodium (Na+) and calcium (Ca++), as shown in Table 3. However, diabetic rats treated with vanillic acid in G3 and those treated with vanillic acid supplemented with AgNPs of vanillic acid in G4 had significantly higher levels of all examined kidney function indices and electrolyte levels. In addition, AgNPs of vanillic acid in G4 significantly improved kidney function and electrolyte levels as compared to vanillic acid in G3.

### 3.5. Immunoglobulins

Table 4 displays that IgA, IgM, and IgG immunoglobulin G2 levels were also elevated in STZ-induced diabetes. However, the levels of these increased immunoglobulins were significantly decreased when diabetic rats in groups G3 and G4 were treated with either vanillic acid or vanillic acid-coated AgNPs. In G4, vanillic acid-coated AgNPs decreased immunoglobulin levels more than the normal vanillic acid.

### 3.6. Pathology

#### 3.6.1. Renal Tissue

**Figure 5:**
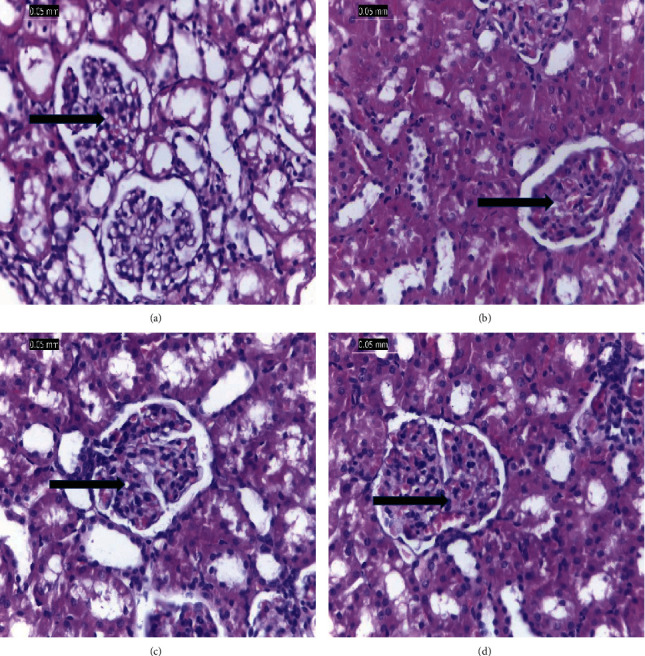
(a) The normal renal tissues of the negative control group (G1). (b) The renal tissues of the diabetic rats (G2) with glomerular ischemia and an inflammatory infiltrate of interstitial mononuclear cells. (c) The kidney of the STZ-induced diabetic rats treated with vanillic acid (G3) with mild tubular atrophy, glomerular ischemia, and interstitial inflammation. (d) Minor inflammation or nearly normal renal tissues of G4 treated with vanillic acid-coated AgNPs (arrows show glomerulus) (H&E, ×400).

The renal tissue of G1 is shown in Figure 5(a) with normal renal tissue, blood vessels, interstitial tissues, renal tubules, and living epithelium. In Figure 5(b), the renal tissues of STZ-induced diabetic rats show reduced vascular tufts, tubular shrinkage, inflammatory interstitial mononuclear infiltration, and glomerular ischemia.  Figure 5(c) shows that STZ-induced diabetic rats treated with vanillic acid (G3) led to the regeneration of renal tissue despite mild tubular atrophy, glomerular ischemia, and interstitial inflammation. In addition, G4 rats (STZ-induced diabetic rats treated with vanillic acid-coated AgNPs) show renal tissues appearing almost normal or with minor signs of inflammation (Figure 5(d)).

#### 3.6.2. Pancreas

**Figure 6:**
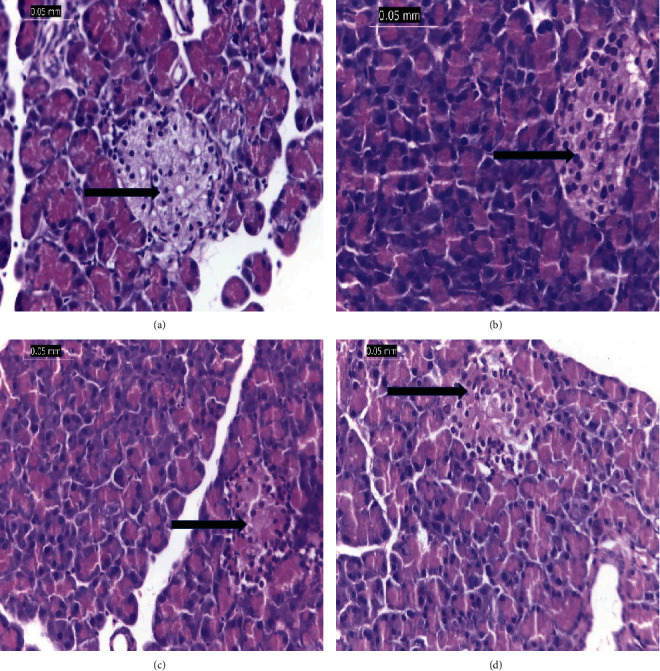
(a) The pancreatic tissue of the negative control. (b) The pancreatic tissues of the STZ-induced diabetic rat (G2) display significant inflammation and infiltrated corroded large ducts. (c) The pancreatic tissues of the STZ-induced diabetic rat (G3) treated with vanillic acid display a mild inflammatory infiltrate around large ducts. (d) The pancreatic tissue of the diabetic rats treated with vanillic acid-coated AgNPs displays nearly normal appearance. Arrows show islet of Langerhans (H&E, ×400).

Figure 6(a) shows a typical pancreatic cell of G1 rats with acini and a typical islet of Langerhans. The pancreatic tissues, on the other hand, were extensively damaged by the STZ injection; pancreatic tissue of G2 is shown in Figure 6(b), which shows inflammation of the corroded main channels as well as the acini, lymphocytes, and eosinophils between the islets. In G3 (Figure 6(c)), the pancreatic tissue shows mild inflammatory infiltration around large ducts. Additionally, in G4 (Figure 6(d)), the pancreatic tissues of rats treated with vanillic acid-coated AgNPs did not exhibit any signs of inflammation in the islets or around big ducts; as a result, these tissues are essentially identical to those found in the normal pancreas of the negative control group (G1).

## 4. Discussion

AgNP-coated vanillic acid was successfully synthesized and showed a surface plasmon resonance (SPR) peak at 450 nm which is consistent with earlier studies [10, 11]. Additionally, the dynamic light scattering (DLS) analysis revealed that the vanillic acid generated with AgNP coating was, on average, 52.3 nm in size. This result is in line with Vivek et al. [21], Zhang et al. [20], Alamri et al. [5], and El Rabey et al. [10].

Diabetes induction in the positive control group by STZ increased FBS, HbA1c, and IL-6 [33], whereas vanillic acid treatment exhibited a protective effect in rats with STZ-induced diabetes as revealed in the reduction of the elevated FBS [14, 21]. In addition, treating diabetic rats with vanillic acid and vanillic acid-coated AgNPs had significantly lowered serum levels of FBS, HbA1c, and IL-6 than diabetic rats of the positive control group treated with STZ. Vanillic acid's methoxy group scavenging free radicals is what gives it its medicinal antioxidant and antidiabetic qualities [7–9, 25] in comparison to vanillic acid-coated with silver nanoparticles (AgNPs), which improves the antioxidant and therapeutic action of vanillic acid and speeds up its delivery to the infected tissues; vanillic acid alone was less effective at lowering the elevated levels of FBS, HbA1c, and IL-6 [5, 10, 25]. The protective antidiabetic and antioxidant activity of vanillic acid-coated AgNPs on diabetes in this study increased the antioxidant and antidiabetic activity of vanillic acid by producing AgNPs that have higher efficacy in drug delivery to the infected sites than the ordinary vanillic acid [5, 10].

The decreased renal tissue homogenate GST, SOD, and CAT and higher MDA in the STZ-administered group (G2) are attributed to DNA damage from STZ-generating free radicals that permanently harm the pancreatic islets [24, 33], while MDA was reduced and the antioxidant enzyme was only marginally increased by treatments with vanillic acid-coated AgNPs. Additionally, vanillic acid-coated AgNPs significantly increased the antioxidant enzymes and decreased lipid peroxidation in the kidney tissue homogenate as compared to the positive control group and more than the normal vanillic acid alone. The vanillic acid antioxidant effect is also due to the methoxy group of vanillic acid, which scavenges free radicals, reduces oxidative stress, and subsequently reduces lipid peroxidation [8, 10, 25]. The vanillic acid-coated AgNPs also exhibited increased antioxidant activity, which increased antioxidant enzyme activity and decreased lipid peroxidation [5, 25].

The levels of uric acid, urea, creatinine, and potassium in the blood of the STZ-administered group were increased while sodium and calcium ions were decreased due to STZ's toxic activity on the pancreatic islets, which led to the development of diabetic nephropathy [24, 33, 34]. Diabetes interferes with intracellular and extracellular electrolytes in diabetic patients, resulting in nephropathy, neuropathy, and vascular issues [24, 35, 36]. All renal functions were enhanced by the antioxidant and antidiabetic properties of vanillic acid and vanillic acid coated with AgNPs. This improvement significantly restored kidney function to baseline levels [24, 37, 38]. Vanillic acid-coated AgNPs were also more successful than vanillic acid alone at improving kidney function and electrolyte levels due to the ameliorating effect of AgNP coating [10, 25].

The increase in immunoglobulins in diabetic rats of the current study is consistent with Al-Malki and El Rabey [33] who stated that the increased immunoglobulins (IgA, IgM, and IgG) in the positive STZ-treated group were brought on by STZ administration that increased the oxidative stress produced by the accumulation of free radicals, whereas the improvement in these immunoglobulins after treatment with vanillic acid is due to the antioxidant and free radical scavenging activity of vanillic acid and its AgNP-coating form that was reflected in the improvement [7–9, 25]. The vanillic acid-coated AgNPs improved the immunoglobulin levels more than the uncoated vanillic acid did because the nanosilver nitrate coating enhanced the antioxidant and antidiabetic effects of vanillic acid [7, 8, 24].

STZ injection in the positive control group caused severe pathological effects on the kidney tissues, as shown by the glomerular ischemia with reduced vascular tufts, interstitial mononuclear inflammation, and significant tubular atrophy, which is consistent with the fact that STZ-induced diabetes causes pathological changes to the vital organs [24, 39, 40]. The renal tissue of the STZ-induced diabetic rats, however, was significantly improved following vanillic acid therapy as a result of the antioxidant and free radical scavenging properties of vanillic acid [7, 8, 10]. Additionally, treatment of STZ-induced diabetic rats with silver nitrate nanoparticles of vanillic acid virtually restored their renal tissues to normal with no signs of inflammation because it boosted the antioxidant and antidiabetic activity of vanillic acid [7, 8, 25].

The STZ-administered group also showed an increase in free radicals and oxidative stress, which resulted in pathogenic effects on the pancreatic tissues. These effects included inflammation of the acini, lymphocytes, a visible inflammatory infiltrate, corroded large ducts, and eosinophils between islet cells [33, 39]. STZ-induced diabetes alters crucial organs [24, 38, 39]. On the other hand, when vanillic acid was administered to the diabetic rats, its methoxy group, which has antioxidant and free radical scavenging characteristics, significantly enhanced all altered pancreatic tissues [7, 8, 24]. The silver nanoparticle coating has also improved the antioxidant efficacy of vanillic acid [8, 15, 25, 41].

## 5. Conclusions

Vanillic acid reduced hyperglycemic activity, oxidative kidney injury, and renal failure in diabetic rats. Thus, it was possible to infer vanillic acid's nephroprotective effects in diabetic rats. Vanillic acid's antioxidant properties may be responsible for the consequence, which leads to a disruption in the downstream inflammatory cascade and necroptotic kidney injury. The study also suggests that vanillic and vanillic acid-coated AgNPs are promising new nephroprotective medicines. The use of vanillic acid silver nanoparticles also decreased inflammation and protected against diabetes more effectively than the use of vanillic acid alone, demonstrating improved vanillic acid antioxidant and antidiabetic activity. Vanillic acid-AgNP coating increases its biomedical viability as confirmed by all recent research done in this field by facilitating its delivery to the infected sites than the ordinary vanillic acid molecules.

## Figures and Tables

**Table 1:** Effect of vanillic acid and vanillic acid-coated AgNP treatment on fasting blood sugar (FBS), glycated hemoglobin (HbA1C), and interleukin-6 (IL-6) in male rats with STZ-induced diabetes.

Parameters	Statistical tests	G1 (negative control)	G2 (positive control)	G3 (treated with vanillic acid)	G4 (treated with vanillic acid-coated AgNPs)
FBS (mg/dl)	Mean ± SD	87.0 ± 0.96^d^	281.66 ± 1.83^a^	202.33 ± 2.74^b^	167.66 ± 2.07^c^
	LSD 0.05 = 6.987				
	*T*-test	—	-69.80^∗∗∗^	20.31^∗∗∗^	44.60^∗∗∗^

HbA1C (%)	Mean ± SD	5.03 ± 0.05^d^	7.26 ± 0.09^a^	6.46 ± 0.12^b^	6.00 ± 0.07^c^
	LSD 0.05 = 0.209				
	*T*-test	—	-26.48^∗∗∗^	21.90^∗∗∗^	13.78^∗∗∗^

IL-6 (pg/ml)	Mean ± SD	22.33 ± 0.91^d^	79.00 ± 2.98^a^	60.66 ± 1.47^b^	39.66 ± 0.91^c^
	LSD 0.05 = 3.618				
	*T*-test	—	-32.77^∗∗∗^	13.58^∗∗∗^	18.56^∗∗∗^

Data are displayed as mean (*M*) ± standard deviation (SD). Means with different superscripts (a, b, c, or d) are statistically different at *P* = 0.05, according to post hoc ANOVA analysis. When they have the same superscript, they are not significantly different at *P* = 0.05. LSD: least significant difference; NS: not significant. Each group consisted of 6 rats, and tests were repeated 6 times. ^∗∗∗^Statistically highly significant (at *P* = 0.001).

**Table 2:** Effect of vanillic acid and vanillic acid AgNP treatment on MDA and antioxidant enzymes (CAT, SOD, and CAT) in male rats with STZ-induced diabetes.

Parameter.	Statistical tests	G1 (negative control)	G2 (positive control)	G3 (treated with vanillic acid)	G4 (treated with vanillic acid-coated AgNPs)
GST (U/g kidney tissue homogenate)	Mean ± SD	111.00 ± 3.28^a^	23.33 ± 1.28^c^	74.33 ± 4.10^b^	96.66 ± 4.00^b^
	LSD 0.05 = 11.442				
	*T*-test	—	43.59^∗∗∗^	-9.48^∗∗∗^	-14.79^∗∗∗^

SOD (U/g kidney tissue homogenate)	Mean ± SD	126.00 ± 3.84^a^	44.66 ± 3.39^c^	73.33 ± 1.28^b^	89.66 ± 2.74^d^
	LSD 0.05 = 30.981				
	*T*-test	—	31.71^∗∗∗^	-8.44^∗∗∗^	-9.31^∗∗∗^

CAT (U/g kidney tissue homogenate)	Mean ± SD	3.46 ± 0.11^a^	0.67 ± 0.02^d^	1.43 ± 0.04^c^	2.52 ± 0.03^b^
	LSD 0.05 = 0.215				
	*T*-test	—	23.52^∗∗∗^	-17.52^∗∗∗^	-39.94^∗∗∗^

MDA (nmol/g kidney tissue)	Mean ± SD	0.33 ± 0.01^d^	1.62 ± 0.02^a^	0.90 ± 0.00^b^	0.70 ± 0.02^c^
	LSD 0.05 = 0.051				
	*T*-test	—	-70.65^∗∗∗^	20.95^∗∗∗^	33.37^∗∗∗^

Data are displayed as mean (*M*) ± standard deviation (SD). Means with different superscripts (a, b, c, or d) are statistically different at *P* = 0.05, according to post hoc ANOVA analysis. When they have the same superscript, they are not significantly different at *P* = 0.05. LSD: least significant difference; NS: not significant. Each group consisted of 6 rats, and tests were repeated 6 times. ^∗∗∗^Statistically highly significant (at *P* = 0.001).

**Table 3:** Renal function in diabetic male rats treated with vanillic acid and vanillic acid-coated AgNPs.

Parameters (mg/dl)	Statistical tests	G1 (negative control)	G2 (positive control)	G3 (treated with vanillic acid)	G4 (treated with vanillic acid-coated AgNPs)
Urea	Mean ± SD	22.66 ± 0.42^d^	76.00 ± 1.31^a^	44.00 ± 1.31^b^	37.00 ± 0.73^c^
	LSD 0.05 = 2.009				
	*T*-test	—	-50.59^∗∗∗^	43.81^∗∗∗^	40.36^∗∗∗^

Creatinine	Mean ± SD	0.63 ± 0.02^d^	1.53 ± 0.02^a^	1.10 ± 0.03^b^	0.86 ± 0.02^c^
	LSD 0.05 = 0.075				
	*T*-test	—	-24.64^∗∗∗^	20.55^∗∗∗^	31.62^∗∗∗^

Uric acid	Mean ± SD	3.90 ± 0.09^a^	3.96 ± 0.11^a^	3.9 ± 0.11^a^	3.90 ± 0.20^a^
	LSD 0.05 = 0.437				
	*T*-test	—	-0.33^NS^	0.19^NS^	0.72^NS^

Na+	Mean ± SD	145.66 ± 0.55^a^	117.00 ± 0.73^d^	126.00 ± 0.36c	133.66 ± 1.11^b^
	LSD 0.05 = 1.501				
	*T*-test	—	37.71^∗∗∗^	-24.64^∗∗∗^	-19.76^∗∗∗^

K+	Mean ± SD	4.93 ± 0.05^d^	6.93 ± 0.09^a^	5.73 ± 0.05^b^	5.36 ± 0.02^c^
	LSD 0.05 = 0.208				
	*T*-test	—	-13.69^∗∗∗^	09.48^∗∗∗^	20.61^∗∗∗^

Ca++	Mean ± SD	11.50 ± 0.07^a^	07.22 ± 0.04^d^	08.30 ± 0.07^c^	09.69 ± 0.02^b^
	LSD 0.05 = 0.159				
	*T*-test	—	60.32^∗∗∗^	-19.12^∗∗∗^	-91.64^∗∗∗^

Data are displayed as mean (*M*) ± standard deviation (SD). Means with different superscripts (a, b, c, or d) are statistically different at *P* = 0.05, according to post hoc ANOVA analysis. When they have the same superscript, they are not significantly different at *P* = 0.05. LSD: least significant difference; NS: not significant. Each group consisted of 6 rats, and tests were repeated 6 times. ^∗∗∗^Statistically highly significant (at *P* = 0.001).

**Table 4:** The effect of vanillic acid-coated AgNPs on the blood immunoglobulins (IgA, IgM, and IgG) in male rats with diabetes.

Parameters	Statistical tests	G1 (negative control)	G2 (positive control)	G3 (treated with vanillic acid)	G4 (treated with vanillic acid-coated AgNPs)
IgA (mg/l)	Mean ± SD	103.33 ± 3.65^d^	342.33 ± 4.15^a^	311.33 ± 2.01^b^	281.66 ± 3.90^c^
	LSD 0.05 = 9.865				
	*T*-test	—	-64.49^∗∗∗^	5.08^∗∗∗^	14.22^∗∗∗^

IgM (mg/l)	Mean ± SD	131.00 ± 1.46^d^	350.33 ± 4.76^a^	321.33 ± 2.69^b^	302.00 ± 2.03^c^
	LSD 0.05 = 6.293				
	*T*-test	—	-64.64^∗∗∗^	9.70^∗∗∗^	12.48^∗∗∗^

IgG (mg/l)	Mean ± SD	526.33 ± 02.59^c^	735.66 ± 08.58^a^	750.33 ± 24.45^a^	622.66 ± 01.47^b^
	LSD 0.05 = 35.916				
	*T*-test	—	-28.86^∗∗∗^	-00.84^NS^	11.79^∗∗∗^

Data are displayed as mean (*M*) ± standard deviation (SD). Means with different superscripts (a, b, c, or d) are statistically different at *P* = 0.05, according to post hoc ANOVA analysis. When they have the same superscript, they are not significantly different at *P* = 0.05. LSD: least significant difference; NS: not significant. Each group consisted of 6 rats, and tests were repeated 6 times. ^∗∗∗^Statistically highly significant (at *P* = 0.001).

## Data Availability

All data are available upon request from the corresponding author.

## Abbreviations

AgNPs: Silver nanoparticles
CAT: Catalase
CAS: Chemical Abstracts Service
DLS: Dynamic light scattering
FBS: Fasting blood sugar
G1: A negative control group injection of 0.1 mol/l citrate buffer (pH 4.5)
G2: A positive control group was intravenously injected with freshly prepared streptozotocin (65 mg/kg bw) in a 0.1 mol/l citrate buffer (pH 4.5)
G3: Intravenously injected with freshly prepared streptozotocin (65 mg/kg bw) in a 0.1 mol/l citrate buffer (pH 4.5) and treated with 100 mg/g bw vanillic acid
G4: Intravenously injected with freshly prepared streptozotocin (65 mg/kg bw) in a 0.1 mol/l citrate buffer (pH 4.5) and treated with 100 mg/g bw AgNP-coated vanillic acid
GSH: Glutathione reduced
GST: Glutathione transferase
HbA1c: Glycated hemoglobin
IL-6: Interleukin-6
MDA: Malondialdehyde
ROS: Reactive oxygen species
SOD: Superoxide dismutase
SPR: Surface plasmon resonance
SPSS: Statistical Package for the Social Sciences
STZ: Streptozotocin
TEM: Transmission electron microscope.
